# Lipopolysaccharide (LPS)-Induced Biliary Epithelial Cell NRas Activation Requires Epidermal Growth Factor Receptor (EGFR)

**DOI:** 10.1371/journal.pone.0125793

**Published:** 2015-04-27

**Authors:** Christy E. Trussoni, James H. Tabibian, Patrick L. Splinter, Steven P. O’Hara

**Affiliations:** Division of Gastroenterology and Hepatology, and the Mayo Clinic Center for Cell Signaling in Gastroenterology, Mayo Clinic, Rochester, Minnesota, 55905, United States of America; Texas A&M Health Science Center, UNITED STATES

## Abstract

Cholangiocytes (biliary epithelial cells) actively participate in microbe-induced proinflammatory responses in the liver and contribute to inflammatory and infectious cholangiopathies. We previously demonstrated that cholangiocyte TLR-dependent NRas activation contributes to proinflammatory/ proliferative responses. We test the hypothesis that LPS-induced activation of NRas requires the EGFR. SV40-transformed human cholangiocytes (H69 cells), or low passage normal human cholangiocytes (NHC), were treated with LPS in the presence or absence of EGFR or ADAM metallopeptidase domain 17 (TACE) inhibitors. Ras activation assays, quantitative RT-PCR, and proliferation assays were performed in cells cultured with or without inhibitors or an siRNA to Grb2. Immunofluorescence for phospho-EGFR was performed on LPS-treated mouse samples and specimens from patients with primary sclerosing cholangitis, primary biliary cirrhosis, hepatitis C, and normal livers. LPS-treatment induced an association between the TLR/MyD88 and EGFR/Grb2 signaling apparatus, NRas activation, and EGFR phosphorylation. NRas activation was sensitive to EGFR and TACE inhibitors and correlated with EGFR phosphorylation. The TACE inhibitor and Grb2 depletion prevented LPS-induced IL6 expression (p<0.05) and proliferation (p<0.01). Additionally, cholangiocytes from LPS-treated mouse livers and human primary sclerosing cholangitis (PSC) livers exhibited increased phospho-EGFR (p<0.01). Moreover, LPS-induced mouse cholangiocyte proliferation was inhibited by concurrent treatment with the EGFR inhibitor, Erlotinib. Our results suggest that EGFR is essential for LPS-induced, TLR4/MyD88-mediated NRas activation and induction of a robust proinflammatory cholangiocyte response. These findings have implications not only for revealing the signaling potential of TLRs, but also implicate EGFR as an integral component of cholangiocyte TLR-induced proinflammatory processes.

## Introduction

Biliary epithelial cells (cholangiocytes) form a simple epithelial layer that separates the intrahepatic bile duct lumen from liver parenchyma [1]. Cholangiocytes perform the essential role of bile modification and transport of biliary and blood constituents, exist in an environment rich in potential mediators of cellular injury, and participate in portal tract repair processes. Cholangiocytes are periodically exposed to potentially pathogenic organisms or products derived from these microbes [2,3,4]. Indeed, the liver is a major organ for bacteria-derived lipopolysaccharide (LPS) clearance, and, while LPS undergoes metabolism in Kupffer cells and hepatocytes, it is excreted in bile in bioactive form [5,6]. Moreover, in cholestatic liver diseases, including the cholangiopathies primary biliary cirrhosis (PBC) and primary sclerosing cholangitis (PSC), cholangiocytes are exposed to increased levels of enteric microbe-derived LPS [6] through the portal venous circulation. How cholangiocytes respond to this cellular insult and the relevance of this response to hepatobiliary diseases remains to be fully explored.

Toll-like receptors (TLRs), an evolutionarily conserved family of pathogen recognition receptors, are essential for effective innate immune responses following detection of pathogen associated molecular patterns (PAMPs) [7,8]. Human cholangiocytes express multiple TLRs [9,10,11] and several studies have implicated cholangiocyte pathogen recognition receptors in persistent inflammation associated with several cholangiopathies including PSC (TLR4 and TLR9) [1–3]. Cholangiocyte TLRs sense and respond to microbes (e.g., the parasitic protozoan, *Cryptosporidium parvum*) and microbe-derived metabolites including LPS (agonist of TLR4) and lipoteichoic acid (LTA, agonist of TLR2). These potentially injurious stimuli can induce cholangiocyte reactivity, including the secretion of cytokines and chemokines, thus promoting fibroinflammatory processes [11–17]. Using a cell culture model we demonstrated that *Cryptosporidium parvum* and the TLR4 agonist, LPS, induced TLR/Myd88-dependent activation of NFkB [9]. We subsequently demonstrated that LPS (as well as other TLR agonists) induced the rapid activation of NRas, but not other Ras isoforms [12]. Activated Ras promotes a variety of signal transduction cascades including MAPK, PI3K, and RAL-GTPase pathways that regulate cell proliferation, survival, differentiation, and proinflammatory cytokine production [13]. In cholangiocytes, the activation of NRas was essential for robust LPS-induced Interleukin 6 (IL6) expression and proliferation [12]; however, the mechanism by which LPS induces cholangiocyte NRas activation is not known.

Cholangiocytes express the epidermal growth factor receptor (EGFR), an upstream mediator of Ras activation [14]. EGFR has been implicated in growth and repair processes in a variety of epithelial cells [15,16], including growth of normal and neoplastic cholangiocytes [17,18]. EGFR activation typically occurs in response to EGFR ligands, including EGF, Transforming growth factor alpha (TGFα), Epiregulin (EREG), Amphiregulin (AREG), and heparin-binding EGF-like growth factor (HB-EGF). These ligands are expressed as transmembrane proteins that are cleaved from the membrane into the extracellular space where they function as autocrine or paracrine ligands for the EGFR. LPS has recently been shown to functionally transactivate cholangiocarcinoma cell EGFR through ADAM17 (TACE)-dependent release of TGFα [19]. Therefore, transactivation of the EGFR is an important mechanism by which LPS stimulates cholangiocyte inflammatory, proliferative and repair processes, which likely involve NRas/MAPK activation.

In the present manuscript we interrogate the role of TACE, EGFR, and the downstream molecular adaptor, Grb2, in LPS-induced NRas activation. Our cumulative data suggest that LPS induced NRas activation was sensitive to EGFR and TACE inhibition and correlated with EGFR phosphorylation, suggesting a progressive transactivation of the EGFR. Our data also suggest that ADAM17 (TACE) and the EGFR molecular adaptor protein, Grb2, are required for LPS-induced NRas activation as well as LPS-induced IL6 expression. We further demonstrate that LPS injected into the tail vein of mice promotes robust cholangiocyte EGFR phosphorylation. Finally, we demonstrate that phospho-EGFR is elevated in the chronic cholestatic liver disease, primary sclerosing cholangitis (PSC).

## Materials and Methods

### Ethics Statement

The study was conducted in accordance with the code of federal regulations and written informed consent from the donors was obtained. This study was approved by the Mayo Clinic Institutional Review Board (IRB# 10–005896; Pathobiology of Hepatic Epithelia) and Institutional Animal Care and Use Committee (A61313).

### Cell Culture and LPS or Inhibitor Treatment

H69 cells, a well-characterized, SV40-transformed human cholangiocyte cell line [20] (a gift from Dr. D. Jefferson, Tufts University, Boston, MA), and normal human cholangiocytes (NHC), low passage human cholangiocytes isolated from normal liver [21] (a gift from Dr. Medina, University of Navarra, Pamplona, Spain), were used. When appropriate, cells were incubated in serum-free and EGF-free media or cultured in the presence or absence of inhibitors to EGFR (CAS 879127-07-8, Millipore), or Adam17/TACE (TAPI 1, Millipore) at concentrations of 10μM and 25 μM, respectively. For siRNA depletion experiments, cells were transfected with a siRNA to Grb2 (Ambion) or a scrambled control siRNA (Ambion) using Fugene HD transfection reagent (Promega) as per manufacturer’s instructions. Cells were then treated with 200 ng/ml of LPS (Sigma) for the indicated times.

### NRas Activation Assay

NRas activation was determined as reported previously [12]. Briefly, lysates were cleared, and diluted to equal protein concentration as confirmed by Bradford Analysis. Raf-RBD beads (Cytoskeleton) were added to each sample and incubated at 4°C, collected by centrifugation, washed, and resuspended in Laemmli Sample buffer (Bio-Rad). The samples were then run on a Tris-HCl gel, transferred to a nitrocellulose membrane, and immunoblotted with a primary antibody to NRas (Santa Cruz) and appropriate secondary antibody (LiCor). The membrane was visualized using LiCor’s Odyssey infrared imaging system. Ponceau Red staining for total GST-RBD was used as loading control.

### Immunoprecipitation and Western Blot

Immunoprecipitation was performed on total protein extracted from H69 cells cultured in the presence or absence of LPS. Lysates were pre-cleared with Protein A/G sepharose beads (Santa Cruz) at 4°C and incubated with a MyD88 (Santa Cruz) or Son of sevenless homolog 1 (SOS1; Santa Cruz) antibody overnight at 4°C. Protein A/G sepharose beads were added to the samples, centrifuged, washed in ice-cold PBS, and resuspended in Laemmli sample buffer. Western blots were performed for Grb2 (Santa Cruz) or TLR4 (Imgenex). Western blots were also performed using cell lysates from untreated, siRNA or inhibitor-treated cells that were cultured in the presence or absence of LPS. Samples were run on a 4–15% gradient Tris-HCL gel, transferred to a nitrocellulose membrane, and immunoblotted with primary antibodies for Grb2 (Santa Cruz), EGFR (Cell Signaling), or EGFR pY1068 (Cell Signaling) and appropriate secondary antibody (LiCor). Membranes were visualized on the LiCor Odyssey Infrared imaging system.

### RNA Extraction and RT-PCR

RNA was extracted using Trizol (Invitrogen) following manufacturer’s instructions. RNA was then reverse transcribed into cDNA using the Superscript III kit (Invitrogen), and used as template to perform quantitative PCR using primers specific for IL6.

### Enzyme-linked immunosorbent assay

Culture medium was centrifuged to remove cellular debris, and 100 μl of cleared medium from H69 cells cultured in the presence or absence of LPS was used in the AREG ELISA following the manufacturer's instructions (R&D Systems). ELISAs were read using a 450-nm wavelength against a 630-nm reference wavelength. Concentration of AREG (pg/ml) was calculated using standard curve analyses.

### In Vitro Cell Proliferation Quantitation

Cell proliferation was quantitated using a Nexcelom automated cell counter. H69 cells were plated at a starting density of 5,000 cells/well in 96-well plates. All cells were treated with 200 ng/ml of LPS for 0, 24, 48, or 72 hours in the presence or absence of inhibitors. After each respective time-point, cells were trypsinized and counted.

### Liver tissues

Sixteen liver tissue specimens consisting of 4 PSC, 4 primary biliary cirrhosis (PBC), 4 chronic hepatitis C (HCV), and 4 normal livers from surgical resection or explant were utilized. Diseased specimens fulfilled clinical, serological, histological, and/or cholangiographic criteria for their respective diagnoses and had cirrhotic stage fibrosis. Liver specimens were fixed in 10% neutral buffered formalin, embedded in paraffin, and sectioned (4 μm). For the mouse studies, LPS (5 mg/kg body weight) or saline (control) was injected into the tail vein of C57-black mice. For the *in vivo* EGFR inhibition studies, Erlotinib (3 μM) was injected intraperitoneally one day prior to LPS treatment and daily over the course of 3 days. Mice were sacrificed at 1, 2, and 3 days post-LPS injection, and livers were initially perfused with a 0.9% sodium chloride solution, and perfused with a 4% paraformaldehyde solution to preserve liver samples for paraffin embedding, histological sectioning, and immunofluorescence.

### Immunofluorescence and FRET

H69 cells were cultured in the presence or absence of LPS for the indicated time points, fixed in a 3% paraformaldehyde solution, washed in PBS, permeabilized with a 0.1% Triton X-100 solution, blocked in 5% serum, and incubated overnight at 4°C with primary polyclonal antibody to EGFR pY1068 (Sigma). Cells were rinsed in PBS, incubated with appropriate Alexa Fluor (Invitrogen) secondary antibody for 1h at room temperature, washed, and mounted in ProLong Gold with DAPI (Invitrogen). Tissue sections were deparaffinized, rehydrated in an ethanol gradient, and boiled in antigen unmasking solution (Vector Labs). Slides were incubated in a blocking solution containing 10% FBS, 1% BSA, and 0.1% Triton-X 100 for 1h at room temperature, incubated with primary antibody to EGFR pY1068 or proliferating cell nuclear antigen (PCNA, PC10, Santa Cruz Biotechnology), washed with a 0.1% Tween-20 solution in PBS, incubated with Alexa-Fluor secondary antibody, washed, and mounted with Prolong gold with DAPI. Confocal immunofluorescence microscopy was performed with a Zeiss LSM 510 as previously described [22]. Adobe Photoshop CS3 (Adobe Systems, San Jose, CA) was used to quantitate fluorescence intensity [23].

Acceptor photobleach Förster Resonant Energy Transfer (FRET) analysis was performed on H69 cells grown on glass coverslips transfected with a Myc-tagged Grb2 plasmid in the presence or absence of LPS for the indicated time-point. Cells were fixed in a 3% paraformaldehyde solution, rinsed in PBS, and blocked in a solution containing 5% serum, 5% glycerol, and 0.04% sodium azide for 45 minutes at room temperature. Cells were incubated with an anti-MyD88 antibody and anti-Myc-tag antibody at 4°C, washed and incubated with a Cy3 or Cy5-conjugated secondary antibody for 45 minutes at room temperature. Coverslips were rinsed in PBS and once in dH20. Coverslips were mounted on the slides using dH20. The acceptor fluorophore (Cy5) was photobleached in a region of interest and the intensity of Cy3 (donor) fluorescence was measured. The FRET efficiency for both conditions (estimate of the fraction of energy transfer to the acceptor per donor excitation event) was calculated using the Donor_max_/Donor_min_ method [24].

## Results

**Fig 1:**
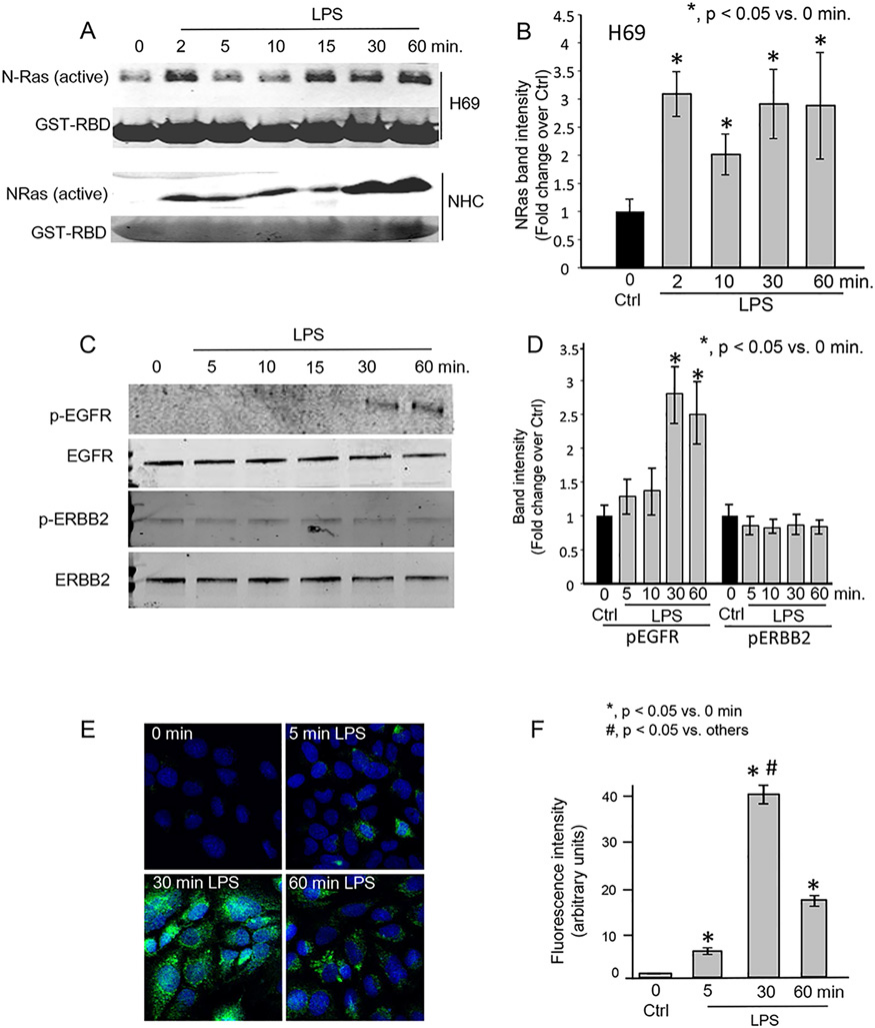
LPS induces NRas activation in cultured human cholangiocytes. A. Confluent NHC and H69 cells were treated with LPS for 0, 2, 5, 10, 15, 30, and 60 minutes. LPS induced rapid and persistent NRas activation. B. Quantitation of NRas intensity. NRas band intensity was normalized to GST-RBD Ponceau red band intensity and is presented as fold change over the 0 time point +/- SEM from three independent experiments. C. Western blots for phospho-EGFR (pEGFR), total EGFR, phospho—ERBB2 (pERBB2), and total ERBB2 were also performed on lysates from the LPS-treated H69 cells. Phospho-EGFR was detected 30 and 60 minutes post-LPS treatment. D. Quantitation of pEGFR and pERBB2. Band intensity was normalized to total EGFR or ERBB2, respectively, and is presented as fold change over 0 time point control. E. Confocal immunofluorescence microscopy detected a progressive increase in phospho-EGFR following LPS treatment of H69 cells. F. Semi-quantitation of fluorescence intensity demonstrates an increase of phospho-EGFR fluorescence through the 30 minute time point. Data is presented as mean +/- SEM from three independent experiments and >100 cells per experiment.

We performed NRas activation assays to demonstrate the time-course of LPS-induced NRas activation. LPS induced rapid (within 2–5 minutes) and persistent (60 minutes) NRas activation in both H69 and NHC (Fig 1A and 1B). Given that i) cholangiocytes express the ERBB family members EGFR and ERBB2, and ii) previous reports demonstrated LPS-induced EGFR phosphorylation in cholangiocarcinoma [17,18,25], we performed western blots for phospho-EGFR or phospho-ERBB2 from 0–60 minutes post-LPS treatment. EGFR phosphorylation was below the detection limit at 0–15 minutes; but evident at 30 and 60 minutes post-LPS treatment; ERBB2 phosphorylation was not detected at any of the time points (Fig 1C and 1D). We also performed confocal immunofluorescence microscopy for phospho-EGFR in H69 cells. At 5 minutes post-LPS treatment, a small proportion of cells exhibited phospho-EGFR (Fig 1E). However, at 30 minutes post-LPS treatment, we observed a robust increase in phospho-EGFR. Quantitation of fluorescence intensity showed a significant increase in phospho-EGFR at 30 minutes post-LPS treatment compared to 0 hour (untreated) sample (Fig 1F). These results suggest that LPS not only induces NRas activation, but EGFR phosphorylation as well.

**Fig 2:**
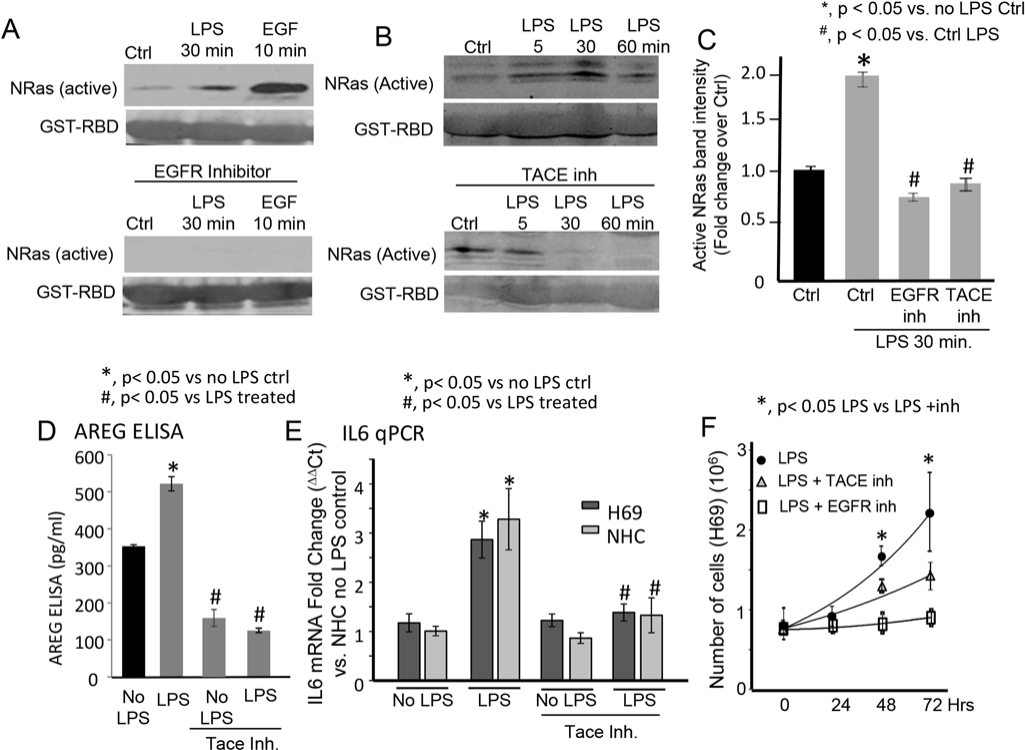
Inhibition of ADAM metallopeptidase 17 (ADAM17 [TACE]) blocks LPS-induced NRas activation, AREG secretion, IL6 expression and cholangiocyte proliferation. A. The EGFR inhibitor blocked LPS-induced NRas activation at the 30 minute post-LPS treatment time point. The EGFR inhibitor also blocked EGF-induced NRas activation. B. The TACE inhibitor, TAPI-1, blocked LPS-induced NRas activation at the 30 and 60 minute post-LPS treatment time point. C. Quantitation of NRas immunoblot band intensity for the 30 minute post-LPS timepoint for both the EGFR and TACE inhibitors. Data is presented as fold change +/- SEM from four independent experiments. D. An ELISA demonstrated that the TACE inhibitor (TAPI-1) blocked LPS-induced secretion of the EGFR ligand, AREG. Data is presented as pg/ml from three independent experiments. E. RT-PCR was performed and demonstrated that the TACE inhibitor blocked LPS-induced IL6 expression in both H69 and NHC cells. Data is presented as fold change (ΔΔCt) vs. NHC no LPS control. F. The TACE and EGFR inhibitors also block LPS-induced cholangiocyte proliferation. In the presence of LPS, H69 cells exhibited a doubling time of 46 hours, while TACE inhibitor treated cells exhibited a doubling time of approximately 80 hours (p < 0.05 at 48 and 72 hours control vs. inhibited cells). EGFR inhibitor treated cells exhibited minimal proliferation over the course of the experiment. Data is presented as mean number of cells +/- SEM from three independent experiments.

We previously demonstrated that LPS-induced activation of NRas is TLR4-dependent, yet TRAF6-independent [12]. We therefore asked whether LPS-induced NRas activation required EGFR or TACE. NRas activation assays demonstrated that both the EGFR inhibitor and TACE inhibitor (TAPI-1) blocked LPS-induced NRas activation at the 30 minute time point (Fig 2A, 2B and 2C). We next asked whether LPS could induce the release of EGFR ligands to the cell culture media. While TGFα was not detected by ELISA in control or LPS treated cells (data not shown), LPS treatment induced, in a TACE sensitive manner, the release of the EGFR ligand, AREG (Fig 2D). TACE inhibition also suppressed LPS-induced IL6 mRNA expression (Fig 2E). Moreover, both TACE inhibition and EGFR inhibition suppress LPS-induced cholangiocyte proliferation (Fig 2F). These results support that LPS induces NRas activation and IL6 expression through TACE-dependent EGFR activation.

**Fig 3:**
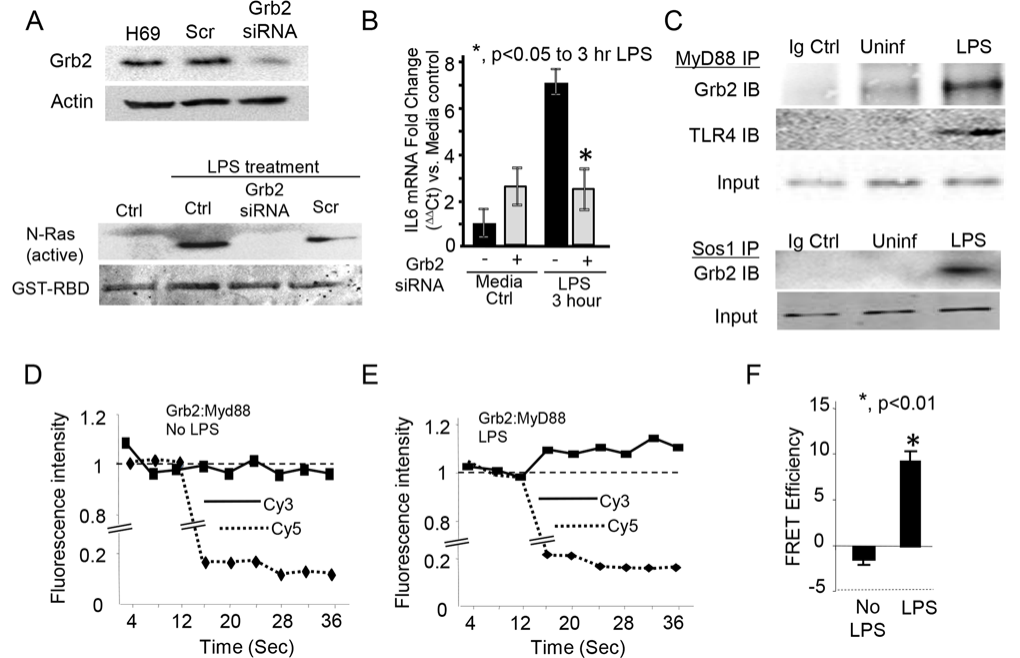
Depletion of the downstream EGFR molecular adaptor, Grb2 blocks LPS-induced NRas activation. A. H69 cells were treated with either a scrambled control (Scr) or a Grb2 siRNA. Expression of Grb2 was normalized to actin to demonstrate efficient depletion. Untransfected (Ctrl), control scrambled (Scr) siRNA, and Grb2 siRNA transfected cells were treated with LPS. Grb2 siRNA blocked LPS-induced NRas activation. B. Quantitiative PCR demonstrated that Grb2 siRNA blocked LPS-induced IL6 expression. The experiment was performed in triplicate; *, p<0.05 compared to LPS treatment in the absence of the siRNA. LPS also induced an interaction between components of the TLR4 and EGFR signaling complex. C. Co-immunoprecipitations were performed on cells cultured in the presence or absence of LPS. Myd88 was immunoprecipitated from lysates of cholangiocytes cultured in the presence or absence of LPS. LPS induced an increase in the amount of Grb2 immunoprecipitated with MyD88, and an interaction between MyD88 and TLR4. Moreover, LPS induced an interaction between the well characterized Ras guanine exchange factor, Sos1, and its molecular adaptor, Grb2. Acceptor photobleaching quantitative FRET further demonstrated LPS-induced Grb2 and MyD88 interaction. D. In the absence of LPS, photobleaching of the acceptor Cy5 (at 16s) did not induce an increase in donor fluorescence, indicating that these molecules are greater than 10 nm apart. In contrast, LPS-treatment induced an increase in donor Cy3 fluorescence detection following Cy5 (acceptor) photobleaching (E), demonstrating the induced proximity (≤ 10 nm) of MyD88 and Grb2. F. FRET efficiency was calculated using the Donor_max_/Donor_min_ method. The LPS-treated cells demonstrated energy transfer between the fluorophores (~10%), while the cells cultured in the absence of LPS did not.

Having demonstrated that inhibition of either EGFR or the known mediator of EGFR activation, TACE, could prevent LPS-induced NRas activation, we next assessed whether depletion of the downstream mediator of EGFR-dependent NRas activation, Grb2 was required for LPS-induced NRas activation. RNA-interference was used to selectively knock-down Grb2 and we again performed an NRas activation assay. LPS-induced NRas activation was blocked in cells depleted of Grb2 (Fig 3A); moreover, depletion of Grb2 blocked LPS-induced IL6 expression (Fig 3B). Together, these results suggest that the EGFR-Grb2 complex is required for the LPS-induced activation of NRas and LPS-induced cholangiocyte expression of the proinflammatory mediator, IL6. Having demonstrated that depletion of the Grb2 molecular adaptor diminishes LPS-induced NRas activation and IL6 expression, we assessed whether Grb2 interacts with components of the TLR4 signaling apparatus. Initially, we immunoprecipitated the TLR adaptor protein, MyD88, from lysates of cholangiocytes cultured in the presence or absence of LPS; we then performed immunoblots for Grb2 and TLR4. The immunoprecipitations demonstrated that LPS induced an interaction between MyD88 and TLR4 as well as MyD88 and Grb2 (Fig 3C). Furthermore, we demonstrated that LPS induced the interaction between Grb2 and the well-known Grb2-associated Ras guanine nucleotide exchange factor, son of sevenless homolog 1 (SOS1) (Fig 3C). As a complimentary approach to address protein complex formation, we force-expressed a Grb2-Myc construct in cholangiocytes and performed acceptor photobleach FRET analysis on cultured cholangiocytes in the presence or absence of LPS. Overexpressed Grb2-Myc and endogenous MyD88 were detected by confocal microscopy using primary antibodies against Myc-tag and Myd88 followed by secondary antibodies conjugated to Cy3 (donor) and Cy5 (acceptor), respectively. LPS treatment induced an increase in donor Cy3 fluorescence detection following Cy5 (acceptor) photobleaching, demonstrating the LPS-induced proximity of MyD88 and Grb2 (Fig 3D and 3E). The FRET efficiency for the LPS-treated cells demonstrated energy transfer between the fluorophores (~10%) (Fig 3F); further suggesting the induction of a TLR/MyD88: EGFR/Grb2 signaling platform.

**Fig 4:**
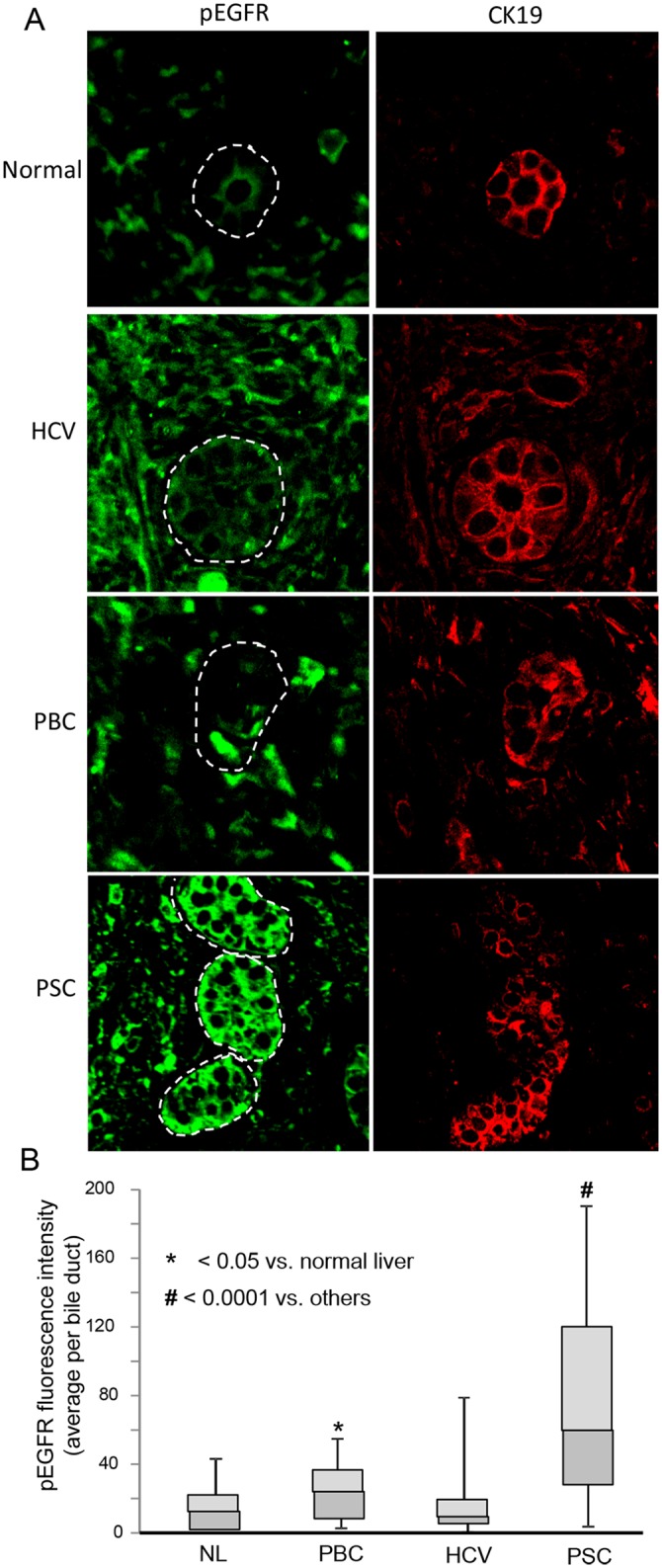
Cholangiocytes in PSC diseased livers exhibit increased phospho-EGFR compared to normal and disease control livers. A. Representative confocal immunofluorescence images for phospho-EGFR (pEGFR) and the cholangiocyte marker cytokeratin 19 (CK19) in normal (NL), HCV-infected, primary biliary cirrhosis (PBC), and primary sclerosing cholangitis (PSC) human livers. Normal livers exhibited minimal phospho-EGFR fluorescence. Cholangiocytes from both PBC and PSC diseased livers exhibited an increase in phospho-EGFR immunofluorescence detection. B. Semi-quantitative analyses of fluorescence intensity, using the Mann Whitney U test, revealed an increase in phospho-EGFR immunofluorescence in both PBC (p < 0.05) and PSC (p < 0.0001) compared to normal livers. Moreover, cholangiocytes from PSC diseased livers exhibited an increase in phospho-EGFR immunofluorescence compared to all other conditions (p < 0.0001). Data is presented as box plots (N = 4 for each condition; minimum of 5 ducts per sample) with mean (horizontal line between shaded boxes), 25^th^ and 75^th^ percentiles, with bars indicating maximum and minimum values.

**Fig 5:**
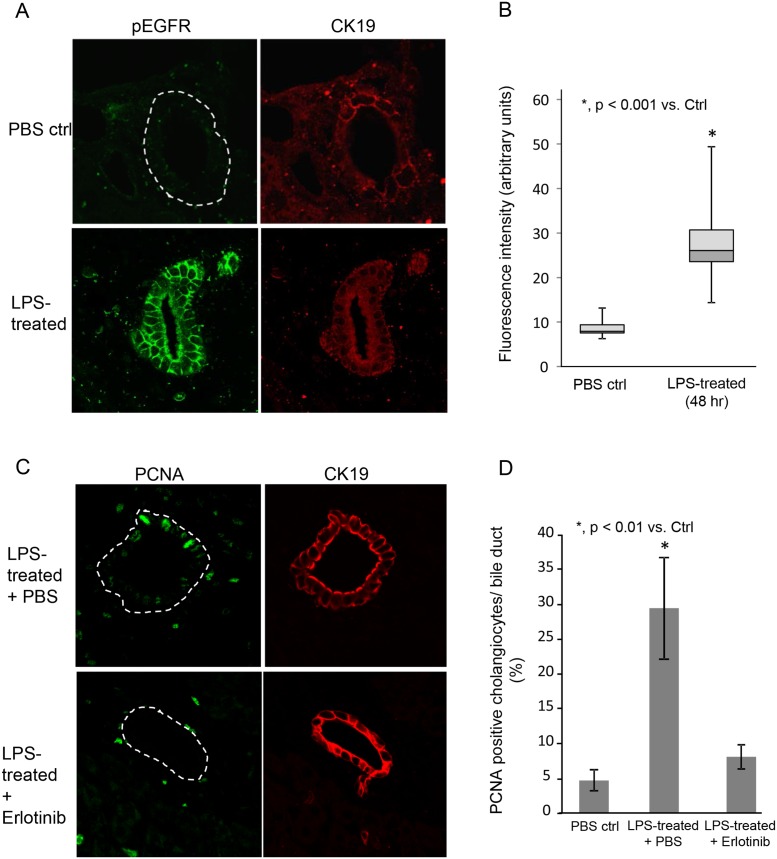
LPS induces cholangiocyte EGFR phosphorylation and proliferation in a mouse model. LPS (5mg/kg body weight) or saline (control) was injected into the tail vein of C57-black mice and the mice were sacrificed at 48 hours post-injection. A. Representative confocal immunofluorescence images for phospho-EGFR and cytokeratin 19 (CK19) in PBS control and mice treated with LPS. Control livers exhibited minimal phospho-EGFR fluorescence. Cholangiocytes from LPS-treated mouse livers exhibited an increase in phospho-EGFR immunofluorescence detection. B. Semi-quantitative analyses of fluorescence intensity, using the Mann Whitney U test (p < 0.001). Data is presented as box plots (N = 3 for each condition; minimum of 4 ducts per sample) with mean (horizontal line between shaded boxes), 25^th^ and 75^th^ percentiles, with bars indicating maximum and minimum values. C. Representative confocal immunofluorescence images for PCNA and CK19 in LPS treated and LPS treated with in the presence of the EGFR inhibitor Erlotinib. Cholangiocytes from LPS treated mice in the absence of Erlotinib exhibited increased PCNA positive nuclei. D. Quantitative analysis of PCNA positive nuclei. The number of PCNA positive cells per bile duct was counted (N = 3 for each condition; minimum of 4 ducts per sample) and is presented as a percentage of total cholangiocytes.

We previously demonstrated that PSC cholangiocytes exhibit increased NRas activation compared to disease control and normal livers [26]. Having demonstrated here, in a cell culture system, that LPS-induced NRas activation requires the EGFR, we determined using confocal immunofluorescence microscopy, whether phospho-EGFR was present in cholangiocytes of livers with hepatobiliary disease. In normal liver, phospho-EGFR was restricted primarily to the apical plasma membrane in cholangiocytes and exhibited weak fluorescence. In contrast, the cholangiocytes from primary sclerosing cholangitis (PSC) livers exhibited elevated phospho-EGFR, with an approximately 5-fold increase vs. normal (NL), primary biliary cirrhosis (PBC) and Hepatitis-C virus (HCV) infected livers (Fig 4A and 4B). We further addressed whether LPS induced cholangiocyte EGFR phosphorylation and proliferation in a mouse model. LPS (5mg/kg body weight) was injected into the tail vein of C57-black mice and the livers were assessed for EGFR phosphorylation and proliferation by confocal immunofluorescence. Control saline injected animals exhibited minimal EGFR phosphorylation, while LPS treated animals exhibited robust EGFR phosphorylation (Fig 5A and 5B). Moreover, control saline injected animals exhibited minimal cholangiocyte proliferation, while proliferation was increased in LPS treated animals. The observed proliferation was blocked when the animals were treated concurrently with the EGFR inhibitor, Erlotinib (Fig 5C and 5D). Collectively, these findings: i) directly demonstrate that the EGFR is significantly more activated in PSC cholangiocytes compared to normal liver and other liver diseases; ii) indirectly support the concept that cholangiocytes may contribute to the biliary inflammation and fibrosis observed in PSC, and iii) demonstrate that LPS can induce EGFR phosphorylation and proliferation in an animal model of hepatic injury.

## Discussion

**Fig 6:**
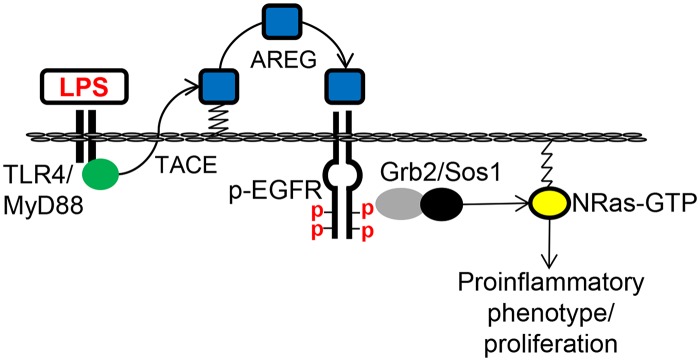
Working model of LPS-induced cholangiocyte NRas activation. We propose that TLR4 activation by LPS promotes TACE-dependent cleavage of the EGFR ligand, AREG. Cleaved AREG engages the EGFR promoting EGFR phosphorylation and recruitment of the molecular adaptor, GRB2. GRB2 and associated guanine nucleotide exchange factor, SOS1, then promotes GTP-bound NRas, and subsequent signal transduction for the expression of proinflammatory mediators and cholangiocyte proliferation.

We previously demonstrated that PAMPs initiate NRas activation in a TLR-dependent manner [12] and NRas activation contributed to the proinflammatory phenotype of LPS-treated cholangiocytes *in vitro*. In the present manuscript we interrogated the mechanisms of NRas activation. The main findings are that: i) LPS induced NRas activation requires EGFR and TACE; ii) TACE inhibition prevents LPS-induced cholangiocyte IL6 expression and proliferation; iii) a downstream mediator of EGFR activation, Grb2, is essential for LPS-induced NRas activation; iv) cholangiocytes from PSC livers exhibit increased EGFR phosphorylation; and v) mice treated with LPS exhibit increased cholangiocyte EGFR phosphorylation and proliferation. The findings reveal the signaling potential of TLRs, and implicate the EGFR as an integral component of cholangiocyte TLR-induced proinflammatory and reparative processes (Fig 6). Moreover, our data support that EGFR is phosphorylated in PSC, a progressive, incurable cholangiopathy characterized by biliary tract inflammation and fibrosis [27].

Not only is the biliary tract periodically colonized by microorganisms [2,3], microbially-derived molecules are delivered directly to the liver through enterohepatic circulation. Hence, it is not surprising that the cells of the liver, including cholangiocytes, sense and respond to endogenous (e.g. bile acids, nucleotides) and exogenous (e.g. microbially-derived) molecules [6,28] which may be potentially injurious and contribute to chronic hepatobiliary diseases. We propose, and our data suggest, that pathogen recognition initiates a signaling cascade that promotes growth factor receptor activation, and this cascade is essential for the cholangiocyte proinflammatory and proliferative response to PAMPS. While cholangiocyte pathogen recognition-dependent NFkB activation induces secretion of innate immune response-associated molecules [9,29], our current results extend the repertoire of TLR-dependent responses to include the activation of a strong mediator of cellular growth and repair, the EGFR.

While a healthy intestine prevents the translocation of microbes and microbially derived products, disruption of the intestinal barrier function results in the translocation of large amounts of these products. Intriguingly, PSC is frequently associated with inflammatory bowel disease (IBD). Indeed, IBD (most often ulcerative colitis) is found in approximately 75% of PSC patients [30,31]. The relationship between IBD and PSC remains obscure, yet forms the basis for the “leaky gut” hypothesis. This hypothesis suggests that the PSC—IBD association may be related to enterohepatic circulation of gut-derived molecules and possibly facilitated by increased intestinal permeability [32,33]. Several observations support the importance of enterohepatically circulated microbial molecules and the cholangiocyte response to microbial products in the etiopathogenesis of PSC. Indeed, freshly isolated cholangiocytes from PSC-diseased livers exhibit hypersensitivity to LPS and other PAMPs [34]. Moreover, PSC is associated with portal bacteremia, bacterial colonization of the bile ducts [35], detection of bacterial 16s ribosomal deoxyribonucleic acid in bile [36,37], and cholangiocytes in PSC livers exhibit an accumulation of LPS [38]. Accumulating evidence now suggests a potentially important role for the intestinal microbiota, and enterohepatic circulation of microbial derived molecules, as a putative mechanistic link between PSC and IBD and a central pathobiological driver of PSC.

Transactivation of the EGFR is a well described phenomenon and occurs through the activation of a variety of plasma membrane receptors including G-protein coupled receptors [39,40], tumor necrosis factor receptor [41], and more recently, pathogen recognition receptors [19,42]. Recently, it was demonstrated that LPS induced the transactivation of cholangiocyte EGFR through TACE-dependent mechanisms [19]. Moreover, it was demonstrated that LPS induced a delayed (~6 hour) EGFR phosphorylation event in cholangiocarcinoma cells which was implicated in LPS-induced cell invasion. Similarly, our data suggest that LPS induced NRas activation, likely through the transactivation of the EGFR in a process involving TACE. Moreover, we implicate EGFR and the molecular adaptor Grb2 in NRas activation.

Cholangiocytes are the target of a diverse group of biliary disorders of different etiologies, collectively referred to as the cholangiopathies [27,43]. The cholangiopathies have in common a number of cholangiocyte-associated processes that may contribute to their pathogenesis, including pro-inflammatory and proliferative responses. Importantly, we demonstrated that the EGFR is highly phosphorylated in cholangiocytes of PSC livers compared to normal, PBC, and HCV livers. PSC is a chronic, cholestatic, fibroinflammatory cholangiopathy with unknown etiology and no effective therapeutic approaches [44]. We recently demonstrated that LPS induced cholangiocyte senescence and the senescence—associated secretory phenotype, likely through NRas activation. Moreover, cholangiocytes in PSC livers exhibit increased NRas activation and a high proportion of senescent cells [26]. Whether the EGFR is involved in LPS-induced cholangiocyte senescence remains unknown but is an active area of investigation. Moreover, patients with PSC are at increased risk of developing cholangiocarcinoma (CCA), a cancer arising from cholangiocytes. Several studies have demonstrated increased expression and activating mutations of EGFR in human CCA [45,46,47,48]; however, this is the first report, to our knowledge, to address the activation state of the EGFR in PSC. Moreover, circulating and intrahepatic LPS are frequently elevated in chronic liver disease. Whether patients with PSC have a genetic predisposition to aberrant LPS-induced EGFR signaling cascades, and how the termination of this signaling cascade is regulated, remains to be determined.

In summary, we have extended our previous findings regarding LPS-induced NRas activation and defined a signaling axis involving the TLR complex and growth factor receptor signaling. This signaling axis is required for robust expression of proinflammatory mediators and proliferation in response to microbially-derived insult. In line with previously reported work, we propose that the EGFR and NRas signaling axis are a central component of fibroinflammatory signaling. Further work is required to better define the molecular pathways and pathophysiological consequences of the TLR/EGFR/NRas signaling axis.
